# Revisiting GUD: An empirical test of the size-dependency of patch departure behaviour

**DOI:** 10.1371/journal.pone.0204448

**Published:** 2018-09-27

**Authors:** Francesco Cozzoli, Giovanna Ligetta, Fabio Vignes, Alberto Basset

**Affiliations:** Laboratory of Ecology, Department of Biological and Environmental Sciences and Technologies, University of the Salento, S.P. Lecce-Monteroni, Lecce, Italy; University of Adelaide, AUSTRALIA

## Abstract

Behaviour related to patch resource exploitation is a major determinant of individual fitness. Assuming the size-dependency of patch departure behaviour, model-based approaches have shown size-mediated coexistence in systems of competing species. However, experimental evidence for the influence of body size on patch use behaviour is scarce. In this study, we explore whether allometric principles provide an underlying framework for interspecific patterns of resource use. To this end, we propose a meso-cosm approach using three species of gastropods differing in size as a model system and ^32^P radio-isotopic techniques as a measure of resource use. Foragers of different size were placed in an artificial patch, provided with a limited amount of labelled resource and let them free to move as resources decrease and scarcity is sensed. We investigated the extent to which individual body size affects the exploitation of resources by examining Giving Up Density (GUD), Giving Up Time (GUT), resource absorption rate and exploitation efficiency as components of individual exploitation behaviour. To compare positive, constant and negative individual size scaling of population energy requirements, experimental trials with an equal numbers and equal biomass of differently sized foragers were carried out, and an experimental trial with equal metabolic requirements was simulated. We observed clear size dependency in the patch departure behaviour of the experimental organisms. Even under conditions of equivalent overall population energy requirements, larger foragers decided to leave the resource patch earlier and at a higher density of resources than smaller ones. Smaller foragers were able to prolong their presence and make more use of the resources, resulting in an inverse body-size scaling of resource exploitation efficiency.

## Introduction

The acquisition of resources is a major determinant of vagile organisms’ ecology and behaviour. It involves decisions regarding where to search, when to feed, which food types to consume and when to terminate feeding [1,2,3,4,5]. An optimal foraging strategy serves to maximize the resource acquisition under the constraints of the environment [6]. Short-term feeding goals [7] can be achieved by following criteria such as maximising the rate of net energy intake [8,9], minimising the variance in the rate of net energy intake [10,11,2,7] and minimising the time spent foraging [3].

Quantitative evaluations of an animal's foraging decisions are commonly obtained by measuring their Giving-Up Density (GUD), i.e. the amount of residual resources remaining in a patch after a foraging episode [12]. GUDs may depend on food densities in other accessible patches (i.e., marginal value theorem [8,13]) and may vary among foraging locations in accordance with other factors associated with the quality of the resource patch [12,14]. The GUD framework traditionally incorporates patch departure determinants such as the finding and processing of resources (*e*.*g*. handling and digesting) (*e*.*g*. [15]), predation risk (*e*.*g*. [16]) and missed opportunities elsewhere (*e*.*g*. [13]), as well as thermoregulatory costs, food toxin costs, the effect of water, knowledge of the territory, risk of injury and competition costs (see [17] for a comprehensive review).

Although the link between patch departure behaviour and individual energetic requirements has been theoretically described [18,19,20], few empirical studies have sought to explore the variation in the energetic characteristics of foragers [17]. These studies mainly address issues such as satiation, forager development, physiology, health and reproductive state (Bedoya-Peréz et al. 2013 [17] and references within). A systematic empirical approach to studying the influence of individual energy requirements on patch departure behaviour has not yet been fully developed.

In this study we explore the use of individual body size as a general predictor of patch departure behaviour. Foragers size is expected to have have a wide-ranging influence on GUDs because of it affects individual’s resource requirements [21,22,23], perception of resource patchiness and density [18,20,24] and home range extension [20]. Larger foragers have higher energetic requirements per unit of time, and they must therefore maintain higher ingestion rates [22]. However, the foragers’ ingestion rates decrease with resource density [25,26]. As the resource approaches depletion, larger foragers are expected to perceive resource scarcity sooner than smaller foragers because of their higher basal energy costs [24]. Consequently, they are expected to abandon the patch at a higher resource density [27,28]. The fractal nature of both environmental landscapes and resource distribution is also expected to lead to body size constraints on resource perception: smaller species may sample their habitat at a finer-grained scale than larger species and thus detect more resources [18,20]. It is hypothesised that differences in individual size may give rise to coexistence mechanisms with regard to a single resource on a multi-patch scale [29,27,30].

Despite some empirical evidence [31,32,33,34], the hypothesis of a positive correlation between forager size and GUD has not yet been tested. Higher GUDs for individuals with higher individual body masses and metabolism have occasionally been observed in studies comparing different species [12,35,36], but contrasting evidence has also been found [37].

Here, we describe an experimental test of whether size-related constraints on the patch behaviour of vagile species are consistent with existing theoretical inferences, i.e. whether differences in GUD and GUT can arise in a homogeneous landscape, where the foragers’ individual energy requirements (approximated as individual body size) are the only variable considered. The GUT, GUD, and trophic transfer of resources of differently sized foragers were therefore measured under controlled conditions. To this end, we used a meso-cosm-based experimental set-up that excluded behavioural changes on the part of foragers in response to both environmental cues such as temperature [38,39], risk perception [40,41,42], resource quality [43,15,44,45], habitat [45,46] and patch structure [47]. The experimental design also excluded inter-specific competition [48,49], but included the effect of intra-specific competition [50,51]. As model foragers we used three common, co-occurring species of temperate freshwater and transitional water gastropods, strongly differing in individual adult body size: *Galba truncatula* (Müller, 1774), *Bithynia tentaculata* (Linnaeus, 1758) and *Ecrobia ventrosa* (Montagu, 1803). To quantify the resources exploited by the grazing gastropods, the microbial and fungal biomass on which they fed was labelled with ^32^P, while GUT was quantified by direct observation. By relating changes in patch departure behaviour to the energy requirement of the foragers, we open a venue to the formulation of general expectations about changes in resource availability, carrying capacity and species coexistence as response to changes in population metabolism and size structure.

## Materials and methods

### Experimental design

In order to test individual behaviour with respect to resource exploitation, we used a two-patch model with two interconnected arenas. Foragers were placed (and let free to move) in one arena containing a limited amount of trophic resources and left them free to feed and move while the other empty arena was intended as a collection patch, where the foragers could move after foraging. Foragers are assumed to be unaware of being constrained in a confined environment with no other resource available except what is provided by the experimenter. Thus, it is expected that they will move to search for more profitable patches as resources decrease and scarcity is sensed. Individual resource exploitation was estimated by radio-isotopic techniques with ^32^P as a marker. Experiments were carried out on three differently sized species of freshwater gastropods (in descending order of size, *G*. *truncatula*, *B*. *tentaculata* and *E*. *ventrosa*.), under two sets of conditions: one with a constant ratio of number of individuals to resources (numeric equivalence) and the other with a constant ratio of biomass to resources (biomass equivalence). Equivalent numbers and biomasses of differently sized foragers have different energy requirements due to the allometric scaling of individual metabolic rates with size. A given number of smaller individuals is expected to have a lower overall energy demand than the same number of larger individuals. However, considering that the size scale exponent of individual metabolic rates is lower than 1, a given biomass of smaller individuals is expected to have a larger overall energy demand than the same biomass of larger individuals [22]. To disentangle the effect of individual and population requirements on resource exploitation, a third condition, i.e. equivalence of population energy requirements (energy equivalence), was modelled from experimental data (Table 1). Overall, this experimental design made it possible to investigate individual size dependency in patch departure behaviour by comparing positive, constant and negative individual size scaling of population energy requirements.

**Table 1:** Table of treatments (see Experimental procedures). Each treatment was repeated for four independent data series. For each of the four data series, the behavioural descriptors of *E*. *ventrosa* and *B*. *tentaculata* at metabolic equivalence (indicated by the asterisk) were obtained by interpolation. The 95% Confidence Intervals of the metabolic rates estimated from Brey’s empirical model [52] account for error propagation from average individual size estimates.

Species	N of Ind.	Individual Size	Individual metabolic rate	Total biomass	Overall metabolic rate
	N	mg AFDW ± 95% CI	mJ day^-1^± 95% CI	mg AFDW± 95% CI	mJ day^-1^± 95% CI
*Galba truncatula*	4	12.88±2.54	5.25±2.16	51.52±10.16	21±8.64
	4			31.08±11.96	14 ±5.68
*Bithynia tentaculata*	6*	7.77±2.99	3.49±1.42	46.62±17.94	21 ±8.52
	7			54.39±20.93	24.43±9.94
	4			1.48±0.20	1.24±0.44
	12			4.44±0.60	3.72±1.32
*Ecrobia*. *ventrosa*	66*	0.37±0.05	0.31±0.11	20±3.00	21±7.26
	79			29.23±3.95	24.49±8.69
	145			53.65±7.25	44.95±16

* Metabolic equivalence, modelled values

### Model organisms

As model organisms we used three differently sized species of temperate freshwater gastropods: *Galba truncatula* (Müller, 1774), *Bithynia tentaculata* (Linnaeus, 1758) and *Ecrobia ventrosa* (Montagu, 1803). They share a common habitat: slow-running freshwaters such as low-velocity rivers, and standing-water bodies such as lakes and marshes [53]. They have similar preferences for calcium-rich waters and alkaline pH [53,54,55]. The three species differ considerably in size: on average, the dry weight of adult *G*. *truncatula* individuals is double that of *B*. *tentaculata* and 30 times that of *E*. *ventrosa*. [56]. These species have been used as model organisms in previous meso-cosm experiments on resource use and absorption [57,28,58] and they are known to easily adapt to laboratory conditions.

Freshwaters gastropods are known to be prevalently active during the daytime [59,60,61] and to feed on leaf detritus as scrapers [57,62]. The dead biomass in detritus consists mostly of structural carbohydrates and poorly decomposed materials. Scrapers access this resource mainly by scratching off the microbial decomposers growing on the detritus surface [57,62]. Based on the literature on *E*. *ventrosa* [63], *B*. *tentaculata* [58] and (closely related to *G*. *truncatula*) *Radix peregra* [58], an average microbial biomass assimilation efficiency of 70% was assumed. *E*. *ventrosa* grazes on microbial biomass by ingesting sediment grains or by scraping the detritus surface [57,63,55]. *G*. *truncatula* and *B*. *tentaculata* also preferentially feed by scraping the microbial biomass from the detritus surface [53,28], but they may adopt alternative feeding strategies when such high-quality resources are not available. By analogy with closely related species, *G*. *truncatula* may be able to feed unselectively on low-quality detritus, much of which can be broken into pieces by its jaws and radula, further triturated by the gizzard and efficiently hydrolysed by digestive enzymes [58]. In contrast, *B*. *tentaculata* may switch to filter-feeding behaviour by using its gills to filter suspended algae from the water column [53].

### Experimental mazes

The experimental mazes simulated two separate patches (intended as homogeneous portions of space within which an individual may satisfy its resource and space requirements for a limited amount of time only) within a single individual home range. They were made of two plastic Petri dishes (diameter 14 cm), connected by a PVC channel 20 cm long and 2.5 cm wide. Experimental animals travelling at full speed were able to walk the length of the channel in a few minutes. The floor of the channels was carefully aligned with the floors of the Petri dishes so as not to hinder the passage of the crawling animals. The mazes were filled with 250 ml of artificial freshwater following Naylor et al. [64]. A plastic gate placed in the middle of the PVC channel allowed the consumers to leave but not to return to the original resource patch. The setup allowed for frequent monitoring and recording of Giving-Up Times by direct observation.

### Resource conditioning

Feeding on ^32^P-labelled resources, foragers bioaccumulated radioactive charge. This made it possible to accurately quantify the amount of residual resource in the patch and the resource transfer to the foragers by measuring the radioactive charge present in the resource and in the foragers at the end of the foraging period [65].

The trophic resource used in this experiment was leaf detritus of *Phragmites australis*. Leaves were originally collected on a single occasion, and were dried first in the sun and then in the oven for 3 days at 60°C. Leaf disks (17 mm in diameter) were cut from dry leaves, avoiding ribs, and sterilised by autoclaving. The disks were conditioned by gently stirring them in environmental water for 17 days at 18°C in aerated tanks. The nutritional quality of the leaves is known to increase during conditioning because of microbial colonisation and the assimilation of nutrients from the water by fungi and bacteria [66,67]. Uptake of radionuclides by the leaf disks also occurs mostly via fungal biomass sequestration [68].

Radioactive labelling was performed by placing the leaf disks for seven days in four aerated flasks, each one inoculated with 200 ml of ^32^P solution having a starting charge of 0.75 μCi ml^-1^. Each of the four resulting sets of labelled disks was considered a unique set of resources and used in a different series of experimental runs. The day before the experiment, the level of ^32^P in each leaf disk was measured by Geiger-Müller counter and over-conditioned disks were discarded.

### Experimental procedures

*E*. *ventrosa and G*. *truncatula s*pecimens were field-collected in April from the Giammatteo channel (Lat: 40.4486, Long: 18.2323, Puglia, Italy). *B*. *tentaculata* specimens were collected at the same time from the nearby Campolitrano channel (Lat: 40.3669, Long: 18.3231). The authorization for the sampling of specimens in the Giammatteo channel was issued by the competent University of Salento. The Campolitrano channel is of public property and no specific permission was required to collect the experimental animals at that location. The species involved in this study are not endangered or protected. The animals were allowed to acclimatise in the laboratory for 2 weeks at 18° C. During acclimation, the animals were fed the same type of leaf detritus used in the experiment. One day before the experiment, all the animals were fasted, measured and weighed *in vivo*, and only individuals belonging to the species’ modal size classes were selected.

The experiments were designed so as to ensure (i) a constant ratio of number of individuals to resources (numeric equivalence) and (ii) a constant ratio of biomass to resources (biomass equivalence). For numeric equivalence, we used four individuals of each species. To represent conditions of biomass equivalence, we used a quantity (7) of *B*. *tentaculata* individuals having the same Ash Free Dry Weight (AFDW) as four individuals of *G*. *truncatula* and a quantity (145) of *E*. *ventrosa* individuals having the same AFDW as four individuals of *G*. *truncatula*. In addition, we used a quantity (79) of *E*. *ventrosa* individuals having the same AFDW as four individuals of *B*. *tentaculata*. In addition, a further treatment with 12 *E*. *ventrosa* individuals was used (Table 1).

Four replicate experimental trials were performed per species, each corresponding to 1 set of ^32^P-labelled leaf disks. For each experimental run, 16 ^32^P-labelled leaf disks were placed in one of the Petri dishes (i.e. the resource patch), keeping the other empty. In terms of conditioned leaf biomass, the amount of resources we provided to the foragers is approximatively slightly more than enough to cover to the daily requirements of one individual of *L*. *truncatula*. [69,70]. Considering that our experiments used a minimal number of four individuals and lasted three days, we assume that foragers would perceive resource scarcity in all treatments except those with the lowest number of *E*. *ventrosa*.

At the beginning of the experimental trial, foragers were released on to the resource patch. Each trial had a duration of 96 h. For each experimental series, a randomly selected run was dismantled immediately to determine the leaf disks’ charge at the beginning of the experiment (start control). Another randomly selected run was left ungrazed and dismantled at the end of the experiment to provide a final control. The experiments were performed under a natural light cycle. During the experimental runs, the mazes were kept in an isolated room to prevent the consumers from perceiving predation risks or other kinds of disturbance that could interfere with their behaviour. The departure time of each individual leaving the resource patch was recorded by direct observation at intervals of 30 minutes during the day time and 12 hours overnight. The inconsistency between time intervals in checking trials may generate bias in our observations. However, the species we used are known to be active prevalently during day time [59,60,61] and our direct observations confirmed that only a limited number of foragers moved during the night. Individuals were removed from the maze after leaving the resource patch.

At the end of the experimental trial, all individuals and leaf disks were washed, oven-dried at 60° C for three days and weighed. AFDW was calculated from dry weight using conversion factors provided by the University of the Salento DisTeBA Ecology Lab. Following Costantini and Rossi (1995) [65], animals and leaf disks were placed individually in glass vials and dissolved in 2 ml of Solvable^TM^ (Perkin Elmer) tissue solubiliser for at least 6 hours at 60°C. The levels of ^32^P in animals and leaf disks were measured as Disintegrations Per Minute (DPM) by a Packard 1900 TriCarb liquid scintillation counter after addition of 10 ml of Ultima Gold^TM^ (Perkin Elmer) scintillator to all vials. Counting efficiency was determined with reference to an external standard, and the results were corrected against blank samples. A further correction was made for radiotracer decay. Measured DPMs were finally converted to Ci (1 DPM = 4.556*10^−13^ Ci).

### Statistical analysis

The analysis took account of four descriptors:

- average Giving Up Time (GUT), expressed as the amount of time foragers spent on the resource patch before leaving (h); individuals that did not leave the patch during the 96 h of experimental trial were excluded from this parameter;
- foragers’net ^32^P net accumulation rate, as the accumulated charge during their presence on the resource patch (nCi h^-1^);
- average Giving Up Density (GUD), expressed as the leaf disks’ residual resource charge at the end of the observation period (nCi leaf disk^-1^);
- average resource exploitation, expressed as the ratio (%) of the average charge of the grazed leaf disks to the average charge of the ungrazed (final control) leaf disks.

Patch behaviour descriptors and average individual body sizes were log-transformed in order to be fitted as power laws (i.e. Y = aX^b^) via ordinary least squares regression. For measures expressed as fractions (e.g. patch exploitation) the relationship was linearised using a logit link function (i.e. log(y/(1-y)). Since measures of explained variance as R^2^ are not available for general linearised models, the explanatory power of the latter models was expressed as McFadden’s Pseudo-R^2^.

Individual metabolic rates and relative 95% Confidence Intervals were estimated in accordance with Brey’s empirical model [52], parametrised for satiated surficial, motile gastropods operating at 18°C, assuming an average organism energy density of 21.5 J mg AFDW^-1^ [71]. To perform the calculation, we used the spreadsheet available at http://www.thomas-brey.de/science/virtualhandbook/spreadsheets/index.html. This spreadsheet allows easy estimation of individual metabolic rates based on size, temperature, depth, taxonomy and lifestyle. Error propagation from average individual size estimates was accounted for in the estimation of the metabolic rates’ 95% Confidence Intervals. Estimates of individual metabolic rates *I* (mJ day^-1^) based on Brey’s empirical model [52] scale with individual body size *M* (mg AFDW), with an exponent close to the theoretical expectation of 0.75 (*I = 0*.*69M*^*0*.*79*^). Population metabolic rates were calculated by assuming that the overall energy requirements of an experimental population are equal to the sum of the individual energy requirements [72]. Consequently, the population energy demand per unit of biomass *I*_*tot*_ (mJ mg^-1^ day^-1^) scales with individual size as *I*_*tot*_*~M*^*-0*.*21*^, the exponent being the inverse of the individual relationship (Table 1).

The experiments were designed to meet criteria of cross-species numeric and biomass equivalence. The third level of analysis, equivalence of population energy requirements (*I*_*tot*_*~M*^*0*^), was derived from the interpolation of collected data. To this purpose, the number of *E*. *ventrosa* and the number of *B*. *tentaculata* with the same energy requirements as four individuals of the larger species *G*. *truncatula* (the only abundance level tested for this species) were calculated in accordance with the individual metabolic rates predicted by Brey [52]. For each of the four independent series of replicates, behaviour measurements for patches with different numbers of individuals were used to linearly interpolate the expected value for a number of individuals energetically equivalent to four *G*. *truncatula* specimens (Table 1). All statistical analyses were performed with the free software R 3.3.3 package [73].

## Results

On average, the *E*. *ventrosa* individuals used in this experiment weighed 0.37 mg AFDW [± 0.05 95% Confidence Interval]. The average weight of *B*. *tentaculata* was 7.77 mg AFDW [± 2.99] and that of *G*. *truncatula* was 12.88 mg AFDW [± 2.54] (Table 1). Average individual metabolic rates estimated from Brey’s empirical model (Brey, An empirical model for estimating aquatic invertebrate respiration, 2010) were 0.31 mJ day^-1^ [± 0.11] for *E*. *ventrosa*, 3.49 mJ day^-1^ [± 1.42] for *B*. *tentaculata* and 5.25 mJ day^-1^ [± 2.16] for *G*. *truncatula* (Table 1).

**Fig 1:**
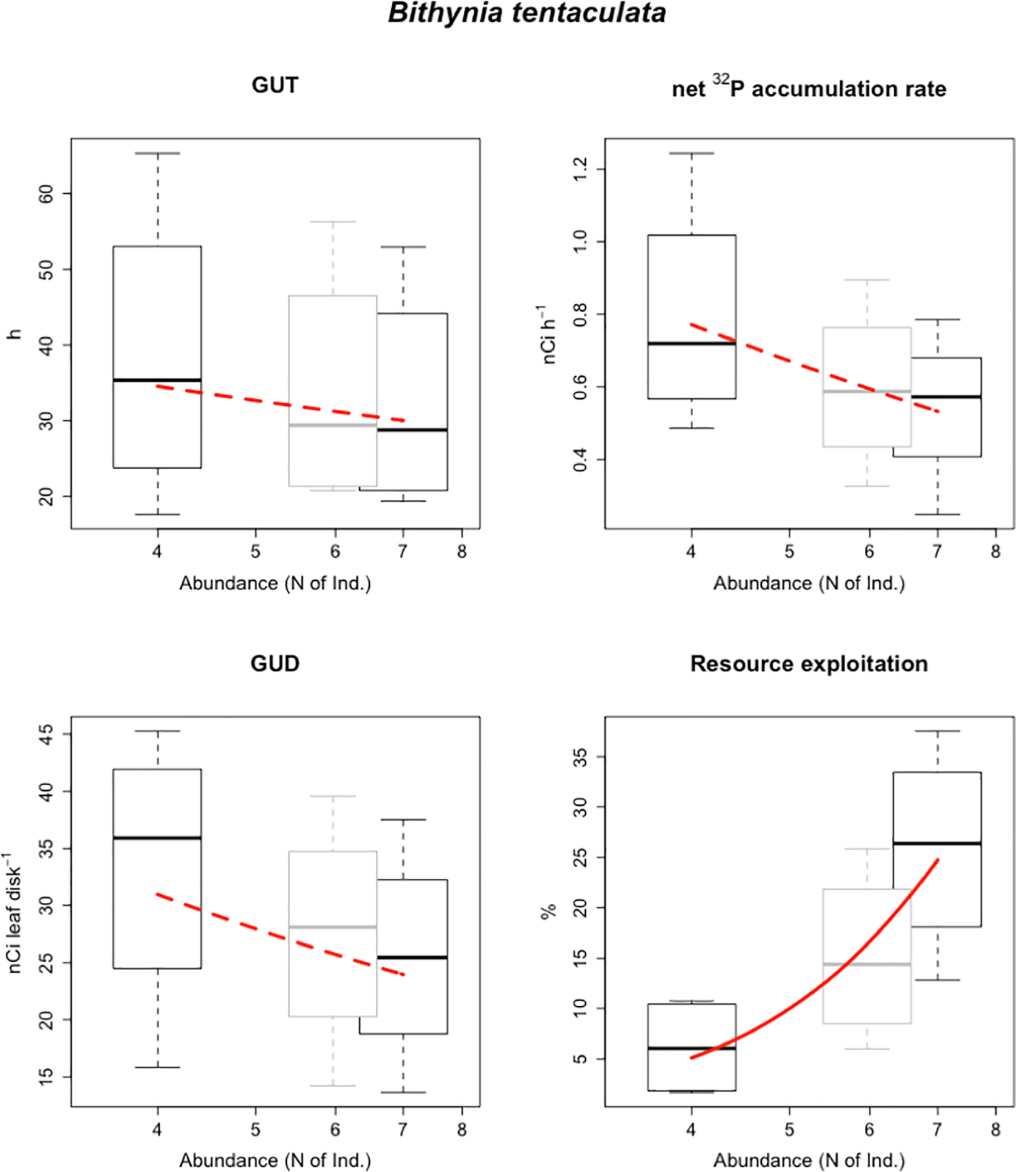
Four descriptors of resource exploitation for increasing numbers of *B*. *tentaculata*: Giving Up Time (h), net ^32^P assimilation rate (nCi h^-1^), Giving Up Density of labelled resource (nCi leaf disk^-1^) and percentage of labelled resource exploited (%). Boxes represent four average values from independent replicates. Red lines refer to the relationships between average species size and exploitation behaviour descriptors modelled as power laws. Red dashed lines refer to non-significant relationships. Grey boxes show the distribution of values (not included in the regression analysis) obtained by interpolating each of the four experimental data series to an *E*. *ventrosa* biomass energetically equivalent to four individuals of *G*. *truncatula*.

All *G*. *truncatula* individuals left the resource patch during the experimental trials, with an average Giving Up Time of 26 h [± 5]). 22% of *B*. *tentaculata* individuals did not leave the patch within the 96 h experimental time. *B*. *tentaculata* individuals that left the patch had an average GUT of 24 h [± 6], with no significant differences between treatments (Fig 1, Table 2). Only 45% of *E*. *ventrosa* individuals left the patch during the experimental time.

**Table 2:** Relationships between *B*. *tentaculata* abundance and resource exploitation descriptors [± 95% Confidence Intervals]: Pearson’s product-moment correlation (R) with associated p-value and parameters of allometric scaling models (Int = Intercept, Slp = Slope), and associated R^2^value.

	R	±95%CI	p	Int	±95%CI	Slp	±95%CI	R^2^
GUT (h)	-0.16	0.70	0.709	49.85	321.04	-0.24	1.52	0.03
Net ^32^P accumulation rate (nCi h^-1^)	-0.45	0.62	0.258	2.54	2.43	-0.26	0.50	0.21
GUD (nCi leaf disk^-1^)	-0.32	0.67	0.44	58.37	292.99	-0.46	1.37	0.10
Resource exploitation (%)	0.81	0.36	0.014	0.06	0.94	3.23	2.13	0.64

**Fig 2:**
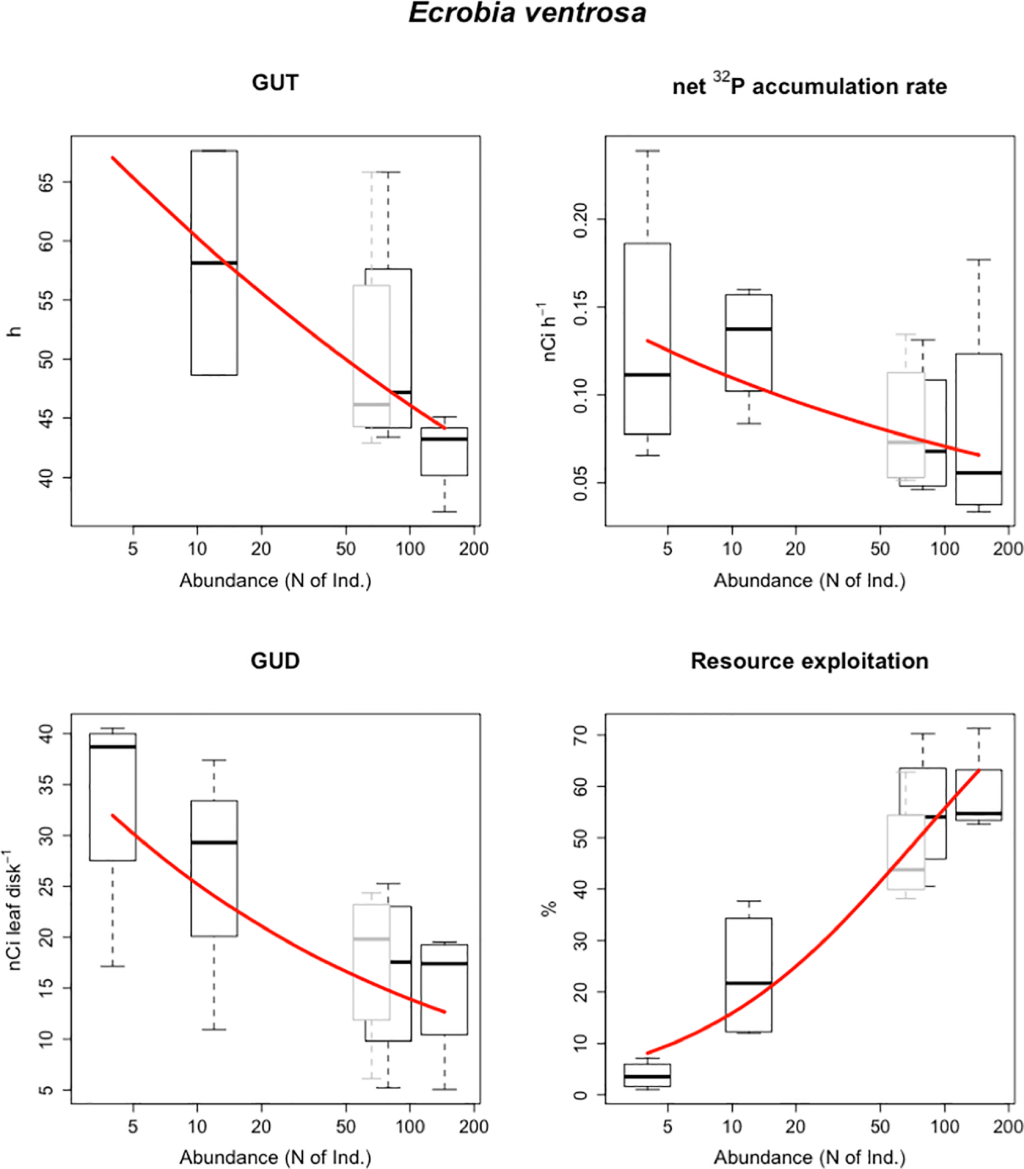
Four descriptors of resource exploitation for increasing numbers of *E*. *ventrosa*: Giving Up Time (h), net ^32^P assimilation rate (nCi h^-1^), Giving Up Density of labelled resource (nCi leaf disk^-1^) and percentage of labelled resource exploited (%). Boxes represent four average values from independent replicates. Red lines refer to the relationships between average species size and exploitation behaviour descriptors modelled as power laws. Red dashed lines refer to non-significant relationships. Grey boxes show the distribution of values (not included in the regression analysis) obtained by interpolating each of the four experimental data series to a *B*. *tentaculata* biomass energetically equivalent to four individuals of *G*. *truncatula*.

Specifically, in the treatments with four individuals (numeric equivalence), only one *E*. *ventrosa* individual out of 16 left the resource patch and this was considered an outlier. For *E*. *ventrosa* it was possible to observe that increases in forager abundance are related to significant reductions in GUT (Fig 2, Table 3), ranging from 58 h [± 18] at an abundance of 12 individuals to 41 h [±5] at an abundance of 145 individuals.

**Table 3:** Relationships between *E*. *ventrosa* abundance and resource exploitation descriptors [± 95% Confidence Intervals]: Pearson’s product-moment correlation (R) with associated p-value and parameters of allometric scaling models (Int = Intercept, Slp = Slope) and associated R^2^ value.

	R	±95% CI	p	Int	±95% CI	Slp	±95% CI	R^2^
GUT (h)	-0.58	0.52	0.101	78.78	52.11	-0.12	0.15	0.34
Net ^32^P accumulation rate (nCi h^-1^)	-0.49	0.40	0.052	0.17	0.13	-0.19	0.19	0.24
GUD (nCi leaf disk^-1^)	-0.59	0.35	0.016	45.69	36.45	-0.26	0.20	0.35
Resource exploitation (%)	0.93	0.09	2e-7	2.74	2.76	0.83	0.24	0.81

**Fig 3:**
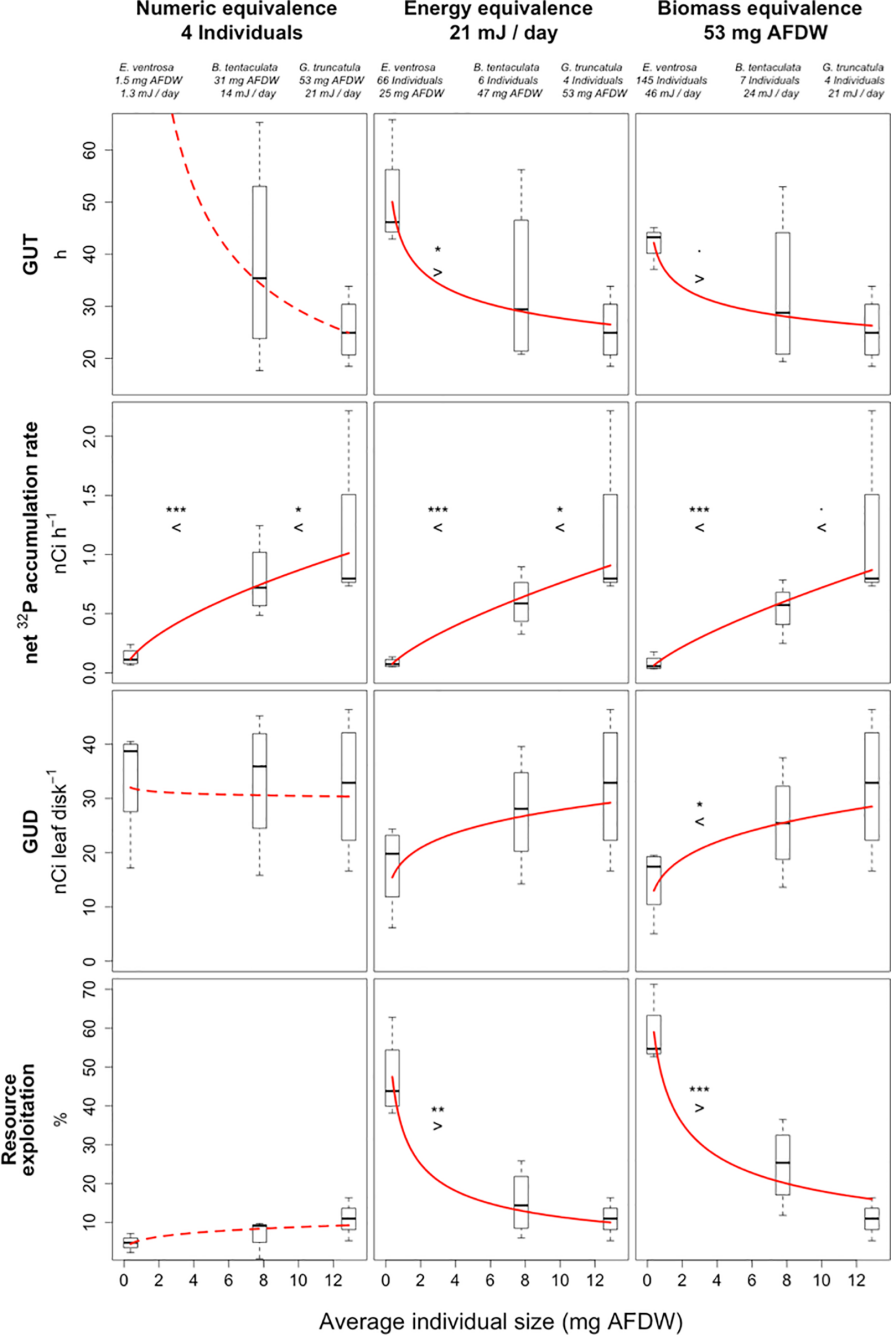
Four descriptors of resource exploitation for numeric, energy and biomass equivalents of three differently sized species of gastropods: Giving Up Time (h), ^32^P net assimilation rate (nCi h^-1^), Giving Up Density of labelled resource (nCi leaf disk^-1^) and percentage of labelled resource exploited (%). Boxes represent four average values from independent replicates. Red lines refer to the relationships between average species size and exploitation behaviour descriptors modelled as power laws. Red dashed lines refer to non-significant relationships.

On average, *E*. *ventrosa* individuals that left the resource patch had a higher GUT than *B*. *tentaculata* and *G*. *truncatula* in conditions of both energy (50 h [± 10]) and biomass (41 h [± 2]) equivalence (Fig 3, Table 4).

**Table 4:** Parameters of the relationships between average individual size and resource exploitation descriptors [± 95% Confidence Intervals] for numeric, energy and biomass equivalents of the largest species *G*. *truncatula*: Pearson’s product-moment correlation (R) with associated p-value and parameters of allometric scaling models (Int = Intercept, Slp = Slope) and associated R^2^ value (Fig 3).

	Equivalence	R	±95% CI	p	Int	±95% CI	Slp	±95% CI	R^2^
GUT	Numeric	-0.01	0.66	0.981	29.16	22.58	0.00	0.32	0.00
(h)	Energy	-0.70	0.35	0.012	41.90	10.92	-0.18	0.13	0.48
	Biomass	-0.57	0.46	0.065	36.96	11.00	-0.13	0.14	0.33
Net ^32^P	Numeric	0.91	0.13	4e-5	0.21	0.08	0.61	0.19	0.83
assimilation rate	Energy	0.94	0.10	1e-5	0.15	0.06	0.70	0.19	0.87
(nCi h^-1^)	Biomass	0.91	0.14	4e-5	0.13	0.06	0.74	0.24	0.82
GUD	Numeric	-0.06	0.57	0.852	31.53	10.96	-0.01	0.17	0.00
(nCi leaf disk^-1^)	Energy	0.54	0.45	0.073	18.44	7.48	0.18	0.20	0.29
	Biomass	0.61	0.41	0.034	16.19	6.61	0.22	0.20	0.37
Resource	Numeric	0.46	0.58	0.211	5.49	3.33	0.22	0.31	0.21
exploitation	Energy	-0.91	0.14	4e-5	33.47	7.80	-0.59	0.21	0.75
(%)	Biomass	-0.92	0.13	5e-5	44.98	8.25	-0.57	0.19	0.80

Regardless of experimental conditions, larger foragers were able to uptake ^32^P at a faster rate than smaller foragers (Fig 3, Table 4). ^32^P net assimilation rates varied from 0.08 nCi h^-1^[± 0.01] for *E*. *ventrosa* to 0.63 nCi h^-1^[± 0.17] for *B*. *tentaculata* and 1.14 nCi h^-1^[± 0.57] for *G*. *truncatula*. Individual size scaling was able to explain more than 83% of variance in the ^32^P net assimilation rate. The estimated scaling exponents are consistent with the theoretical expectation of 0.75 (Table 4). The *E*. *ventrosa* ^32^P net assimilation rate decreased with foragers’ abundance, ranging from 0.13 nCi h^-1^ [± 0.06] at four individuals to 0.08 nCi h^-1^ [± 0.01] at 145 individuals (Fig 2, Table 3).

The average weight of leaf disks at the end of the experimental trial was 14 mg dry weight [± 0.19], with no significant differences between treatments. Ungrazed leaf disks had a final radioactive charge of 31.2 nCi leaf disk^-1^ [± 3.5]. We observed a significant effect of grazing on the residual resource charges (F[7, 489] = 27.64, *p-value* < 0.001), with a maximal decrease of -16.2 nCi leaf disk^-1^ [± 2.1] for the highest abundance of *E*. *ventrosa*. In conditions of numeric equivalence, forager species had no significant effect on resource depletion (Fig 3, Table 4). In conditions of equivalence of population energy requirement (metabolic equivalence), *E*. *ventrosa* had a lower GUD (18 nCi leaf disk^-1^ [± 8]) than the larger *B*. *tentaculata* (27 nCi leaf disk^-1^ [± 10]) and *G*. *truncatula* (32 nCi leaf disk^-1^ [± 12]). In addition, energy-equivalent quantities of *E*. *ventrosa* were able to exploit a higher percentage of resources (47% [± 11]) than *B*. *tentaculata* (15% [± 8]) and *G*. *truncatula* (11% [± 6]). Scaling trends between average forager size and GUD are steepest in conditions of biomass equivalence (Table 4). Biomass-equivalent quantities of *E*. *ventrosa* had a significantly lower GUD (15 nCi leaf disk^-1^[± 7]) and higher exploitation efficiency (58% [± 8]) than *B*. *tentaculata* and *G*. *truncatula*, and exploited more resources than the two larger species (Fig 3). Decreasing GUD and increasing resource exploitation were also observed with increasing *E*. *ventrosa* abundance (Table 2), ranging from 34 nCi leaf disk^-1^ [± 11]) at four individuals to 15 nCi leaf disk^-1^ [± 7] at 145 individuals (Fig 2, Table 3). No significant differences in GUT, ^32^P net assimilation rate and GUD were observed between the two abundance levels of *B*. *tentaculata* (Fig 3, Table 4), possibly due to the limited extent of the abundance gradient over which the observations were made (four *vs*. seven, Table 1).

## Discussion

Overall, we observed clear size dependencies in the patch departure behaviour of the studied model organisms. Larger foragers tended to leave the resource patch earlier and at a higher density of resources than smaller ones. Smaller foragers, able to sustain themselves with lower ingestion rates, had a longer Giving Up Times and a lower Giving Up Densities, and made a more efficient use of resources at the patch level. The observed negative size-scaling of GUT and GUD and positive size-scaling of patch exploitation efficiency are in agreement with both theoretical expectations [27,30,24] and previous empirical evidence [31,33,34].

Given their limited population, in conditions of numeric equivalence (scaling of overall population energy requirements *I*_*tot*_ with individual body size *M*_:_ *I*_*tot*_*~M*^*0*.*79*^), *E*. *ventrosa* foragers may not have perceived resource scarcity in relation to energy requirements, and so did not leave the resource patch. In contrast, *B*. *tentaculata* and *G*. *truncatula*, whose individual energy requirements are from 10 to 15 times higher than *E*. *ventrosa*, tended to perceive resource scarcity more readily and leave the patch within ca. one day. In conditions of equivalent overall population resource requirements (*I*_*tot*_*~M*^*0*^), *E*. *ventrosa* had a longer GUT than larger foragers and was able to consume more resources. This reflects the ability of smaller animals to exploit resources more fully due to their lower individual energy requirements [27]. In conditions of biomass equivalence (*I*_*tot*_*~M*^*-0*.*21*^), the combined effect of higher individual efficiency in resource exploitation and higher population energy requirements enhanced the trend observed in conditions of energy equivalence.

It should be considered that 55% of *E*. *ventrosa* individuals and 22% of *B*. *tentaculata* individuals did not leave the patch during the experimental trial time, while the totality of *L*. *truncatula* did. This pattern supports the interpretation that larger foragers tend to give up earlier than smaller ones. However, it also implies that the GUD of the smaller species may have been overestimated and the exploitation efficiency underestimated. Therefore, the investigated scaling relationships may be characterised by even steeper exponents and higher significance than we estimated.

Consistent with previous observations on aquatic deposit feeders and detritivores [74,75], average species size explains more than 83% of variance in the net ^32^P assimilation rate. Moreover, the observed scaling exponent is consistent with the theoretical expectation of 0.75. Resource ingestion rates are known to decrease with resource density [25,26], and larger foragers may thus reduce their ingestion rates to a sub-optimal level in conditions of resource scarcity [24]. Negative relationships between forager density and the individual amount of foraged resources have often been observed as a result of increasing competition [17]. Furthermore, individuals may adopt compensative responses such as lowering their metabolic rates when they perceive resource scarcity or strong competition [76,77]. In our observations, at increased numerical abundance, *E*. *ventrosa* individuals extracted resources more slowly, indicating that interaction (competition) between individuals has some form of limiting effect on ingestion rates. However, the fact that the observed cross-species size-scaling of the net ^32^P assimilation rate was not significantly different from the null energy expectation of 0.75 could imply that foragers mainly react to resource scarcity by leaving the resource patch earlier rather than by decreasing their ingestion rates.

In our experiments, size scaling explained a larger amount of the observed variance in patch exploitation efficiency (i.e. the ratio of GUD to the amount of resources initially available) than raw GUD. It is thus possible that our experimental organisms adjusted their giving-up thresholds in accordance with the resources available in each replicate, using compensative strategies such as higher GUDs in richer patches [78,79,80,32,81,82]. This may be related to heterogeneity in leaf disk conditioning across experimental series, which generates unexplained variability when the residual charge is considered as an absolute value.

Our observations were conducted on three species only. Although these three species are known to have strong similarities in feeding behaviour, our results may be affected by species-specific resource preferences or the use of alternative pools of resources by *B*. *tentaculata* and *G*. *truncatula* [53,54]. However, it is unlikely that *B*. *tentaculata* adopted filtration feeding during our experiments because we used pre-filtered water. In addition, we did not detect any significant variation in weight between the ungrazed final control leaf disks and those grazed by *G*. *truncatula*, although the highest individual ^32^P net assimilation rate was detected for this species. It can therefore be assumed that *G*. *truncatula* did not feed directly on the leaves but rather that it grazed on the surficial microbial biomass like the other two species. Lastly, the net ^32^P assimilation rate shows that in our experiment, foragers fed on labelled resources at a rate fully consistent with the theoretical expectation of size scaling of individual energy requirements and ingestion rates [22], i.e. as would be expected if all the foragers were exploiting a comparable resource pool.

If the same amount of energy/resources were available for each size class, population density should scale with average population size as the inverse of the scaling exponent of the individual energy requirements, i.e. -0.75 [83]. However, according to theoretical expectations [27,20,24], larger foragers should perceive and exploit a smaller quantity of resources per unit of space than smaller foragers, and indeed this is consistent with our observations. As a result, the size-density relationship should scale with a more negative exponent than -0.75. In contrast with this reasoning, field relationships between macrozoobenthic invertebrate size and the number of individuals foraging on resource patches have been observed to scale with *less* negative exponents (~ -0.5) than the null expectation of -0.75 [84,85], i.e. as if a relatively *larger* quantity of resources was available for large foragers. A potential explanation for these apparently discordant observations is that while small foragers may be more efficient consumers on the single-patch scale, they tend to spend more time in the patch, and they are thus less efficient when searching for new resource patches. Larger foragers, characterised by shorter permanence times and faster ingestion rates, may be more efficient in detecting and acquiring resources on a multi-patch scale. As a consequence, larger foragers may reach a higher density of individuals than what would be expected from purely energy-based considerations.

In addition to size, operational temperature is well known to influence metabolic rates in accordance with the Boltzmann-Arrhenius dependence [22,86,87]. The metabolic dependence of patch exploitation efficiency observed here implies that temperature variations may have an effect on resource patch exploitation efficiency by systematically affecting metabolic rates and individual resource requirements [22,86]. Although our experiments were performed at a fixed temperature, the observed metabolism-based relationship does enable speculative extrapolations regarding the influence of predicted temperature increases on patch exploitation. Increasing temperatures are expected to affect mainly larger consumers by further limiting their ability to exploit resource patches. If this is not compensated by increases in the home range or changes in resource productivity and distribution, we would expect rising temperature to orient selection towards smaller-sized foragers. Supporting this consideration, a large body of observational [88,89] and experimental [90,91,92] research shows that temperature disproportionally affects larger individuals. Future experimental research into temperature variations may help to explain the mechanistic process behind the temperature-size rule and to predict future population and community responses to global warming [93].

Many other factors besides energetics, e.g. habitat characteristics, risks associated with foraging and the type of resources (see Bedoya-Peréz et al. 2013 [17] for a review) are known to influence foragers' patch departure behaviour. However, supporting the hypothesis that patch departure behaviour is to some degree dependent on individual energy requirements (for which body size is a widely accepted proxy), a recent meta-analysis of granivorous rodents' GUDs shows that positive size-scaling of GUD is conserved across a wide range of environmental and taxonomic characteristics, as well as across different types of disturbance to foraging activity [94].

## Conclusion

While much effort has been devoted to investigating the influence of extrinsic factors (e.g. habitat structure and ecological interactions such as predation) on resource exploitation behaviour, the intrinsic influence of forager energetics has yet to be fully addressed in GUD theory [17]. Previous research in this field has mainly focused on contextual and idiosyncratic issues such as satiation and forager development, physiology, health and reproductive state (Bedoya-Peréz et al. 2013 [17] and references within). In contrast, we investigated the systematic variation of patch departure behaviour using body size as a proxy for forager energy requirements. This study therefore frames GUD theory in the broader context of energy scaling research (e.g. the Metabolic Theory of Ecology, [22]). This topic is of particular interest in ecology, because size-dependencies in patch departure behaviour are believed to affect space use, home range size [20,95] species coexistence and resource partitioning [27,30,96]. The results of our experiments can be used to parametrise theoretical models of energetic carrying capacity [97,14], size-related species coexistence [30] and ecological community responses to climate change [93].

## Supporting information

S1 Dataset Averaged experimental data. (XLS)
